# Viral Hepatitis: Retrospective Review in a Canadian Pediatric Hospital

**DOI:** 10.5402/2011/182964

**Published:** 2010-12-29

**Authors:** Paulina Cybulska, Andy Ni, Carolina Jimenez-Rivera

**Affiliations:** ^1^Faculty of Medicine, University of Ottawa, 451 Smyth Road, Ottawa, ON, Canada K1H 8M5; ^2^Clinical Research Unit, Children's Hospital of Eastern Ontario Research Institute, Ottawa, ON, Canada K1H 8L1; ^3^Division of Pediatric Gastroenterology, Hepatology & Nutrition, Children's Hospital of Eastern Ontario, 401 Smyth Road, Ottawa, ON, Canada K1H 8L1

## Abstract

*Introduction*. Clinical presentation of viral hepatitis ranges from mild symptoms to fulminant hepatitis. Our aim is to describe clinical presentation and outcomes of children with viral hepatitis from the Eastern Ontario/Western Quebec regions of Canada. *Methods*. Retrospective chart review of children diagnosed with viral hepatitis at our institution from January 1, 1998, to December 31, 2007. *Results*. There were 261 charts reviewed, only 64 had a confirmed viral etiology: 34 (53%) hepatitis B (HBV), 16 (25%) hepatitis C (HCV), 4 (6.3%) hepatitis A (HAV), 7 (11%) cytomegalovirus (CMV), and 3 (4.7%) Epstein-Barr virus (EBV). Children with HBV presented at a mean age of 6.4 ± 4.6 years. Spontaneous seroconversion (appearance of HBVeAb and loss of HBVeAg) occurred in 21/34 (61.7%). Children with acute hepatitis (HAV, CMV, and EBV) presented with mild abdominal pain, jaundice, and fevers. Overall outcome was excellent. *Conclusion*. Acute and chronic hepatitis in children has a benign course; moreover, HBV spontaneous seroconversion is common in pediatric patients.

## 1. Introduction

Hepatitis in childhood is largely caused by viral etiology. Clinical presentation of acute hepatitis generally includes jaundice and vague abdominal pain, though some children may present with vomiting and fever. These symptoms are reported to be milder and the disease course is shorter in children compared to adults [1]. Children with chronic hepatitis rarely present with symptoms; its prevalence is influenced by country of origin, socioeconomic status, and local outbreaks. 

The incidence of viral hepatitis varies according to geographical areas [2] as well as immunization regime in different countries. The prevalence of hepatitis B in Canadian children has been estimated between 5 and 13 per 100,000 [3]. Approximately 2 billion people have serological evidence of past or current hepatitis B infection in the world; however, global pediatric prevalence is not known [4]. Nearly 90% of viral hepatitis is caused by hepatitis A, B, or C viruses [5]; however, potential hepatotropic viruses that can cause acute hepatitis include Epstein-Barr virus (EBV) and cytomegalovirus (CMV).

 Our aim is to describe clinical presentation and outcome of patients diagnosed with viral hepatitis in the national capital region of Canada.

## 2. Methods

We conducted a retrospective chart review of all patients diagnosed with viral hepatitis including hepatitis A, B, and C, EBV, and CMV from January 1998 to January 2008 at the Children's Hospital of Eastern Ontario.

Demographics included age at diagnosis, gender, and ethnic background [6]. Symptoms at presentation such as jaundice, abdominal pain, vomiting, and fever as well as the need for admission to hospital were recorded. Biochemical parameters included alanine aminotransferase (ALT), aspartate aminotransferase (AST), alkaline phosphatase, gamma glutamyl transpeptidase, bilirubin (conjugated and total), and albumin. Coagulation status was assessed by prothrombin time, partial thromboplastin time, and international normalized ratio. Viral serology included hepatitis B surface antigen (HbsAg), Hepatitis B e Antigen (HbeAg), Hepatitis B surface antibody (HbsAb), Hepatitis B e antibody (HbeAb), Hepatitis A, Hepatitis C serology, EBV, and CMV. Hepatitis B seroconversion was defined as the appearance of HBeAb and loss of HBeAg.

All statistical analyses were conducted using SAS 9.1 (SAS Institute Inc., Cary, NC, USA). Normally distributed continuous variables were summarized using mean and standard deviations. Nonnormally distributed continuous variables were summarized using median and interquartile range. Categorical variables were summarized using frequency and percentage. The percentages of seroconversion in the HBV group were calculated along with an exact 95% confidence interval. The Kruskal-Wallis test was used in the overall comparison of ALT level among the three acute hepatitis groups due to the small number of patients and large differences in the variance of ALT among groups.

## 3. Results

A total of 261 charts were reviewed; however, only 64 patients had confirmed etiology. One patient was diagnosed with herpes simplex virus hepatitis and was not included in the analysis. There were 34 (53%) children with hepatitis B virus (HBV), 16 (25%) with hepatitis C virus (HCV), 4 (6.3%) with hepatitis A virus (HAV), 7 (11%) with CMV, and 3 (4.7%) with EBV. Demographics and clinical presentation of patients with acute hepatitis (HAV, EBV, and CMV) are reported in Table 1. Children with acute viral hepatitis had a benign course; no child had fulminant hepatitis or required liver transplantation. Serology for hepatitis E was not done.

Children with chronic hepatitis were symptom-free and were screened after positive family history. Mean age of children with HBV was 6.4 ± 4.6 years. Ethnic background included Black 11 (32.4%), Caucasian 6 (17.7%), South East Asian 6 (17.7%), Arab 5 (14.7%), Chinese 3 (8.8%), West Asian 2 (5.9%), and Korean 1 (2.9%). Children with an HBV infection had mild elevation of liver function tests with a median AST of 62 IU/mL (interquartile range: 37–113 IU/mL) and a median ALT of 66 IU/mL (interquartile range: 32–105 IU/mL). Nearly one third of patients (29%, 95% CI: 15–47%) with chronic HBV infection had spontaneous seroconversion at the first clinic visit; mean age was 9.9 ± 4.5 years. Another 35% (95% CI: 20–54%) of children seroconverted spontaneously at a mean followup of 5.0 ± 3.5 years (mean age 9.9 ± 3.1 years). Seroconversion rates are reported in Table 2. One patient with HBV was treated with subcutaneous interferon *α* − 2b at 3 years of age for 24 weeks without success. This child spontaneously seroconverted at 7 years of age.

Children with HCV had a mean age of 8.4 ± 4.1 years. Ethnic backgrounds included Caucasian 9 (56.2%), Chinese 6 (37.5%), and Southeast Asian 1 (6.3%). No patient received antiviral therapy against HCV. Elevation of liver enzymes was mild with a median AST of 58 IU/mL (interquartile range: 36–80 IU/mL) and a median ALT of 42 IU/mL (interquartile range: 33–65 IU/mL).

## 4. Discussion

The number of children diagnosed with viral hepatitis with a confirmed etiology was low in our study. One possibility is that a specific serology was not done and children were discharged with a diagnosis of viral hepatitis of unknown etiology. From those who had confirmed viral etiology, we found that only a quarter presented with acute hepatitis related to HAV, EBV, and CMV, and most of them presented with jaundice and abdominal pain. It was found that the elevation of liver enzymes in children with HAV was by far the highest among the three acute hepatitis groups. The CMV group had the lowest elevation of liver enzymes although the overall test did not reach statistical significance, likely due to the small sample size.

The clinical course for children with acute hepatitis was benign, and very few patients needed hospital admission. There were no cases with acute liver failure or need for liver transplantation. Contrary to our findings, Ciocca et al. [7] reported a high incidence of acute liver failure due to HAV in an Argentinean population; this could be explained by disease prevalence and geographical distribution. With the advent of HAV vaccination, mild to fatal cases can be prevented since HAV vaccine has proved efficacious. For instance the incidence of HAV has significantly decreased in the United States after implementing vaccination campaigns [8]. In Canada, vaccination against HAV is currently recommended to high-risk patients or people traveling to endemic areas, autochthones cases are scarce as the incidence of HVA is very low.

On the other hand, infection with EBV can be associated with abnormal liver function tests in up to 80–90% of cases and is often underdiagnosed [9]. It is well known that EBV can also cause lymphoproliferative disorders particularly in children with underlying diseases or postorgan transplantation [10]. It is likely that the number of children with EBV-related hepatitis was underestimated in our study. One can speculate that serology was probably not done. Fortunately, the clinical course of EBV-related hepatitis in immunocompetent patients is benign, and normalization of liver function tests occurs with time.

Similarly, CMV is also a well-recognized cause of hepatitis, particularly in immunocompromised children (i.e., neonates and postorgan transplant) [11]. Seven children had CMV-related hepatitis in our review; all of them were immunocompetent and did not require antiviral therapy. In contrast, Tezer et al. [12] reported the use of ganciclovir for CMV hepatitis in immunocompetent children with excellent response, specifically for those showing progression of the disease. The use of antiviral therapy is not widely recommended and is limited for immunocompromised patients.

Children with chronic viral hepatitis generally have a different course of disease. The majority are symptom-free, and most of them had vertically transmitted infection [13]. The incidence of HBV infection in childhood is not well known and varies geographically. HBV spontaneous seroconversion in our patient population occurred in approximately 65% of children by age 9.9 years. Similar to our findings, Bortolotti et al. [14] reported spontaneous seroconversion in 70% of their children with chronic hepatitis B. The mechanisms for seroconversion are not well known; however, it has been proposed that age at the time of HBV infection, HBV genotype, and host immune system plays a role in this process [15]. Although all patients with HBV infection may present long-term complications such as cirrhosis and hepatocellular carcinoma, those who seroconvert have much lower risk of developing these conditions [16, 17]. Preventive measures such as implementing universal immunization have significantly reduced the incidence of HBV in different countries [18, 19], and Canada has not been the exception. 

Notably, there were more children with chronic HBV than HAV, both potentially preventable conditions with the use of vaccination; this could be explained by the high immigration rate from high HBV prevalent countries and the chronicity it represents as opposed to the acute clinical presentation on children with HAV infection. Approved therapies in the United States for HBV include standard interferon-alpha, lamivudine, and most recently adefovir in adolescents [20]. Success rates vary depending on different factors including viral load and therapeutic agent; however, the overall success rate ranges from 30% to 40%. 

All children with HCV had vertical transmission and were asymptomatic. Elevation of liver enzymes was mild, and no child received antiviral therapy. Mild transaminitis in childhood HCV was reported by Guido et al. [21] where the degree of elevation of liver enzymes strongly correlated to grading of focal necrosis in liver biopsies. Treatment with ribavirin and pegylated interferon-alpha is recommended in some particular cases [22].

In summary, viral hepatitis is frequently seen in our clinical practice; however, most children with a discharge diagnosis of viral hepatitis have no confirmed etiology. Children with acute or chronic viral hepatitis had a benign course and had no long-term complications or need for liver transplantation.

##  Conflict of Interests

The authors have no conflict of interests to declare nor any financial interests with pharmaceutical companies related to this study.

## Figures and Tables

**Table 1:** Acute hepatitis, demographics, and clinical presentation.

Virus (*N*)	Age at diagnosis: years ± SD	Signs and symptoms	ALT (IU/mL, median, interquartile range)	*P* value*
HAV (4)	5.9 ± 1.0	Jaundice, abdominal pain, and vomiting	1791 (739–3830)	.122
EBV (3)	9.7 ± 4.7	Jaundice and abdominal pain	159 (126–196)	
CMV (7)	7.0 ± 6.9	Jaundice, fever, vomiting, and diarrhea	75 (70–260)	

*Kruskal-Wallis test for overall difference among the three groups.

**Table 2:** Spontaneous seroconversion (appearance HBeAb & loss of HbeAg) rate in children with Hepatitis B.

Spontaneous seroconversion	Patients *N* (%, 95% CI)	Age at seroconversion: years ± SD	Time to seroconvert: years ± SD
At first visit	10 (29.4%, 15–47%)	9.9 ± 4.5	N/A
At followup	12 (35.3%, 20–54%)	9.9 ± 3.1	5 ± 3.5
Total seroconversion	22 (64.9%, 46–80%)	9.9 ± 3.7	N/A
No seroconversion	12 (35.3%, 20–4%)	N/A	N/A
